# Clinical Facts and Reflections; Also, Remarks on the Impunity of Murder in Some Cases of Presumed Insanity

**Published:** 1847-04-01

**Authors:** 


### Clinical Facts and Reflections; also, Remarks on the Impunity of Murder in some Cases of Presumed Insanity. By *Thomas Mayo*, M.D., F.R.S., Fellow of the Royal College of Physicians, late Fellow of Oriel College, Oxford; and Physician to the Infirmary of St. Marylebone. Octavo, pp. 217. London, Longman & Co., 1847.

Dr. Mayo has surely not the gift of lucid expression, if the reader may judge from the introductory remarks of his volume.

"The march of science in medical philosophy must follow the laws of the inductive process, and ascend from individual instances to general propositions. In the application of the proposition to general practice, the direction of thought is reversed. But we should err if we limited the science of medicine by this description of it: for *pari passu* with the proceeding there noticed, and subsidiary to the application of the principles so acquired, another highly scientific operation is required. Indeed, the march of science towards the formation and application of general principles will be practically fruitless, if the above-mentioned procedure be deemed all-sufficient; if an inquiry be not at the same time vigorously conducted as to the shades of difference which separate from each other cases of the same kind. In the first point of view in which I have placed this subject, we deal with facts in relation to their common points; but in this second point of view we have to deal with them in relation to their discrepancies. The first operation is statistical in its nature, and would lead to an undiscriminating practice. The latter operation unties the facts thus accumulated, and subjects them to an ordeal which is requisite to their exact use.

"I make the above remarks, in order to obtain its just place and importance for an enumeration of cases, either single or in groups. Thus individualised, they possess perhaps a less philosophical air than in their cumulative state. But their utility is obvious. Indeed, the practical character of the English mind has largely enriched our medical literature with cases contemplated in this point of view. We abound in valuable monographs. I am desirous of adding to their quantity, whatever may be the quality of my contributions." P. 2.

Such is the motive of our author's present appearance before the public. The bulk of his work is taken up with detached, and sometimes fragmentary, reports of cases, deemed to be illustrative of various forms of disease.

We shall briefly notice most of the chapters in succession. Chapter I. is occupied with the history of three cases of Peritonitis.

In the two first, which occurred in children 8 and 11 years of age, purulent effusion was found to have taken place into the abdomen, without, as it would seem, any well-marked symptoms of abdominal suffering having been manifested during life. There is a morbid appearance, related in the account of the dissection of one of these cases, that is surely very uncommon. "The external coat of the peritoneum," it is stated, "the muscles being dissected from it, was red and covered with apparent granulations secreting pus." Are we to understand from this that the external surface of the abdominal peritoneum had been secreting a quantity of pus?

The third case calls for notice only in consequence of the very slovenly and inaccurate manner in which the report has been drawn up. What, pray, is the meaning of the following prescription?

Sumat. pil. Hyd. Chlorid. gr. iv.; Morph. Mur. gr. $\frac{1}{3}$; bis quotidie Olei Ricini, ℥ss. statim. Perstet.

The second Chapter is headed "Enteritic and Gastric Affections with Petechiæ—Petechial Fever." These affections are afterwards called by our author "Petechial Phlegmasiæ." Three examples are related. The second one stands nearly thus. A middle-aged man, subject to dyspepsia, was suddenly seized with pain at the stomach; and shortly afterwards with vomiting. The pulse became weak and faltering; and, within three-quarters of an hour from the first moment of the attack, life was gone. What were the morbid appearances found on dissection?

> "Petechiæ extensively spread between the coats of the stomach, the mucous membrane being perfectly healthy, and there being no appearance of inflammation in the spaces between these petechiæ.
>
> "An ulcer in the posterior part of the aorta, without any aneurismal sac or dilatation, and which had not penetrated the coats of the artery.
>
> "A relaxed and flabby heart, the walls of which tore easily." P. 14.

Now here was a case of serious Organic Disease of the Heart proving rapidly, as it often does, fatal, and in which traces of congestion were found on the coats of the stomach. It must surely require a rare tact for detecting resemblances to find out wherein such a case as the preceding can, in any way, be considered as having aught to do with a "petechial phlegmasia."

In the third case, the symptoms were these. A young athletic man was seized first with vomiting, and then with intense pain at the epigastric and umbilical region, increasing upon pressure. The tongue was clean, and the pulse quiet. Several large petechiæ were to be seen on the arms, and his legs and thighs were covered with them. A blister was applied on the epigastrium, and three grains of calomel were given every six hours, with a saline draught in the intervals. Next day, after the bowels had acted freely, the petechiæ began to fade, and the patient was much better. On the following day, some blood was observed in the stools, and, after a turpentine enema, a very large quantity of blood came away. He rapidly recovered.

Such a case as this is by no means unfrequent. It is well known that many cases of Melæna and Hæmatemesis are connected with a congested state of the Liver; and it has been very reasonably conjectured, by several able physicians, that Purpura is frequently associated with irregularities in the portal circulation. How any one could for a moment entertain the idea that, in such a case, "the petechial state may have constituted the very essence of the disease," seems to us very surprising.

Chap. III. professes to treat of Gastrodynia. The first case related was cured by the use of a Plummer's pill twice a day, continued for several months; the second, by mild alteratives and aperients; and the third, by the muriate of morphia and the extract of opium. The only thing remarkable in the histories of these cases is the unlooked-for announcement by our author, that "each certainly was tending to a fatal termination, when arrested by remedies." Certainly no one could have suspected, from the account given of the symptoms, that they were half so serious. We should have fancied that it would be easy for almost any practitioner to relate scores of similar cases, successfully treated by the most simple and common remedies.

Chapter IV. is occupied with the history of a case of Ileus, accompanied with stercoraceous vomiting, &c., in which the seat of the intestinal obstruction was in the rectum, "of which about ten (the lower) inches were so obstructed as scarcely to admit a quill." It is strange that this obstruction was not discovered during life, more especially as several enemata had been administered. All that we are told upon this score is to this effect:

"We may observe, that the introduction of bougies into the rectum was indicated in this case by the seat of the stricture, and might have relieved it, to the extent of affording a downward passage to the fæces, a tendency towards which had certainly been established by the other means employed, when the case was suddenly and accidentally terminated;" (the patient having died during an act of vomiting, which occurred during sleep).

The history of Dr. Mayo's, and indeed of almost every other, case of obstruction of the bowels, that we read of in the medical journals of the day, only tends to confirm us in the propriety of an opinion which we have for many years entertained; viz. that the treatment, which is generally adopted, oftener does harm than good; and that it would be much better for the patient to be left to Nature's unassisted efforts to relieve herself, than to be drenched, as he is almost inevitably sure to be, with repeated doses of drastic purgatives, which only serve (in a very great many instances) to increase his sufferings and exasperate his disease. If medical men were in the habit of trusting more to the use of leeches—general bleeding also may be necessary—and of continued hot fomentations to the abdomen, and, at the same time, to the frequently-repeated exhibition of purgative enemata, with little or no medicine administered by the mouth, the really remediable cases might in general be promptly relieved; and, should the obstruction arise from an insurmountable cause, there will be the satisfaction of knowing that the remedies employed, although fruitless, have done no mischief.

Chap. V.—The case of Patency of the *foramen Botallii*—which occurred in a tall stout woman who had lived to 57 years of age—is very interesting. She described herself as labouring under an habitual winter cough. She was subject to sudden dyspnœa, obliging her to jump up, if lying down at the time. The pulse was small and quick. The impulse of the heart was strong; the dulness on percussion in the cardiac region was more extended than usual; and there was a loud systolic bruit over the apex of the heart. The patient was labouring under slight Pneumonia, when admitted into the Marylebone Infirmary. No appearance of *cyanosis* was discoverable until four days before death ; and it seemed to have been brought on by the effects of a fright, from the violence of another patient in the same ward. The death was sudden and unexpected.

*Dissection.*—The heart weighed eighteen and a half ounces. The right auricle was large: its muscular substance well developed. The right auriculo-ventricular opening was also large, but tolerably proportioned to the tricuspid valves. These valves were white, and thickened, particularly near the free edges; their columnæ carneæ were remarkably developed. The right ventricle was extraordinarily large, and its walls from three to four-eighths of an inch thick; the columnæ carneæ well marked; the semilunar valves of the pulmonary artery perfect. Its coats, as well as those of the pulmonary veins, greatly distended and thickened, and less supple than usual. The left auricle was large and fleshy, thicker than the right; there was an open communication between it and the right auricle in the situation of the foramen ovale, extending an inch and a quarter from below backwards, and half an inch in the opposite direction. Two membranous bands, extending from the upper to the lower portion of the auricle, divided this opening into three unequal parts. The left auriculo-ventricular opening was normal; the valves ill formed. The under mitral valve almost atrophied; the upper enormously large, about an inch and a half long, and broad in proportion. The left ventricle was greatly hypertrophied; the columnæ carneæ large. The aorta normal.

" The lungs exhibited a few points of semicartilaginous hardness at their summits; they were congested with blood and somewhat œdematous, but everywhere crepitant. The liver large, of the nutmeg character, its weight 64 ounces. The kidneys were small. The other abdominal viscera were normal, as was the brain.

" In this monstrous case of perforate foramen ovale, the patient had attained her 57th year, a strongly made and not unhealthy woman, entirely free from the purple hue which belongs to a circulation thus rendered imperfect. In what way, or by what immediate agency, that arrangement was destroyed which had so long given for the most part a right direction to the respective columns of blood, when the sudden fright incurred by the patient, occasioned an unfavourable turn to her symptoms, and what was the immediate cause of death, are questions of equal difficulty and interest." P. 38.

The above case affords a good illustration of what M. Bouillaud has alluded to (vide page 347 of the present No.) in his remarks on the ætiology of Heart Diseases.

The drift of the three cases of Erysipelas, which are related in the next chapter, is to recommend the use of blisters in the treatment of this disease. Dr. Mayo is of opinion that, by thus substituting another form of cutaneous inflammation for the existing morbid one, very marked benefit may in general be obtained. He alludes to no other local application. His remarks are far from being satisfactory.

The observations on "Intermittence or Periodicity as a character of certain Maladies not of an Agueish origin" in Chapter VII., suggest some useful reflections.

"It has been judiciously observed, in regard to intermittents in which the affection of a given organ is more strongly marked than those symptoms which characterise the disorder as an intermittent, that the affection thus evolved or brought out by the miasma of ague may require a special treatment distinct from, or even at variance with, that which its simple periodicity would suggest. In this point of view the supposed affection may be less appropriately called a masked intermittent, than a tendency to phlegmasia brought into an active state as often as the system is disturbed by the paroxysms of the intermittent. This disturbance, however, if of an inflammatory nature, may not terminate with the paroxysm except in appearance; but the system may be brought gradually into a condition in which the intermittent type may be lost, and a continuous disorder substituted.

"The same discriminate tact is wanted here as in another equally indefinite disorder—Hysteria, in which a phlegmasia may easily combine itself with the nervous affection; a view of the subject, I apprehend, far more frequently applicable than that suggested by the terms simulating, proteiform, &c., often used as descriptive of hysteria. The disorder which this hypothesis represents as simulated is really existent, and must receive its specific treatment, modified by the judgment of the practitioner as he may best use it.

"The above remarks may claim some importance in reference to the present state of intermittent diseases in our country. Ague has declined in frequency. But may we not in some degree exaggerate the effect of presumed improvement in drainage and cultivation of land as the cause of this diminution? Is it not in some degree a change in the form of an endemic?

"The frequently intermittent type of neuralgic disorders, their general susceptibility of benefit from those remedies which are specific in agues, is well known. But it is scarcely conceivable that so great a change should have been effected in the features of periodical disease as the substitution of head-aches, or an epileptic seizure, or a nervous pain, for a rigor, with its subsequent febrile accession, without grounds being at the same time afforded for a careful revision of our practice under these varied circumstances. Nor is this consideration weakened by the remark of Dr. Macculloch, in his valuable work, that the use of the most energetic remedies is in the neuralgic intermittent precisely the same as intermittent fever." P. 59.

The remarks on "Pseudo-Rubeola," in Chapter IX., do not appear to require particular notice, as it seems very doubtful whether the exanthematous eruption in some of the recorded cases had anything to do with Measles. Nor does the practice, that was adopted in some of them, seem very commendable. Dr. Mayo's chief error consists in doing too much. Witness the following

*Case of Cerebral Excitement occurring in an Hysterical Patient.*—"Miss H. was a florid full-grown young lady, aged 20, of the mixed nervous and sanguine temperament. When I was called in, she had for some days laboured under cerebral symptoms; since, indeed, an inefficient period occurred, in the course of the preceding week, acute pain in the head had increased upon her, upon which delirium supervened, with daily rigors and extreme intolerance of light. She had been freely purged in the last three days; nothing further had been done: but her complaints of pain in the head were incessant. I directed that her hair, which was luxuriant, should be cropped close, evaporating lotions applied in reference to heat of surface, the head being often hot, and three grains of calomel every fourth hour. In forty-eight hours the evacuations had become mercurially green. No increase of symptoms had occurred, but no abatement, and her strength had fallen much.

"Having observed some periodicity in her symptoms, and some other hysterical phenomena, and trusting that the calomel had placed her in a state of safety relatively to the congestive part of the case, I now gave her Quinæ. Disulphat. gr. ij. every fourth hour for five doses. On this her strength increased, and the rigors ceased; her mind also became clear. And now set in a series of hysterical symptoms." P. 87.

Here nearly two scruples of Calomel appear to have been given in the course of 48 hours; and this active mercurial course was immediately followed by liberal doses of Quinine. We strongly suspect that a much simpler plan of treatment would have been both wiser and safer. In this case, as in so many others of Hysterical suffering, the bowels were evidently much at fault; for we read that, "ever since the first head-symptoms gave way, the patient had constantly complained of much tenderness of the abdomen. This continued long, and left her gradually, assisted apparently by aperient enemas with assafœtida. Indeed, the symptom so frequent in hysteria shewed itself largely, that, namely, of immense fecal evacuations coming away as the spasmodic symptoms yielded. When waywardness occasionally occurred, we repeated the cold affusion, provided there was present sufficient heat of skin. But she continued long in a very weak state."

This case is related in the chapter on "Hysteria and the Hysterical Diathesis." The object of this chapter is to shew that inflammatory and other morbid affections, occurring in hysterical patients, require the same mode of treatment that is called for under other circumstances.

After the narrative of some cases in the way of illustration, and a criticism upon one reported by Sir B. Brodie in his work on "Various Forms of Local Hysterical Affections," our author observes:

"Much loose language respecting the proteiform nature of hysteria, and its tendency to simulate other diseases, has been led to by the confusion of disease with diathesis, above illustrated. But I am disposed to think, admitting the objectionable nature of such terms, that more valuable practical results would be gained from an hypothesis of palsy simulating hysteria than from the (present) reverse hypothesis. Cases of both kinds may be found in the valuable posthumous medical writings of Dr. Parry, of Bath; and Dr. Billing has recorded a case, in which globus hystericus with palpitation, ensued in a young man upon a violent strain of the spine, resulting from a fall under a heavy weight. I have known no instance in which the treating a 'local hysterical affection' as humoral or inflammatory, would have been so mischievous, as the contemplating that globus hystericus, and the accompanying affection, in the light of hysteria." P. 100.

But then there is infinitely less chance of the latter, than of the former, mistake. For a single case of the one, there are many hundreds, nay thousands, of the other. Again, surely few physicians will agree with Dr. Mayo, that "more valuable practical results would be gained from an hypothesis of Palsy simulating Hysteria than from the reverse hypothesis."

We have said that Dr. M.'s practice appears to be often far more active than the symptoms seem to require. In the chapter on "Bloodletting in cases of Congestion," we meet with the case of an infant, in whom hepatisation of both lungs was believed to have supervened upon an attack of Measles. Now, what was the treatment pursued in this instance? Besides three leeches and a blister to the sternum, the infant appears to have taken daily from three to six grains of Calomel with several grains of Hemlock, for ten days or a fortnight before its death!

Dr. Mayo, at the close of this chapter, quotes with approbation the following extract from the posthumous work of Dr. Parry; telling us, at the same time, that his own experience perfectly coincides with the doctrine taught therein, in its yet more extended application to bleeding for Cerebral Congestion.

"*Difference of effect between small and large bleedings in Hemiplegia.*—When Dr. H., who was about 70 years of age, was seized with hemiplegia, in which he totally lost the voluntary power of his arm and leg, I ordered him to be cupped. This was done only a few hours after the seizure. While the operation was performing, when only four ounces of blood had flowed, the power of voluntary motion returned in his limbs; *but again vanished by the time ten ounces had been taken.*"*

Yet in one of the cases, which he had related, just before, without any comment, the enormous (surely we may say, the excessive) quantity of between 50 and 60 ounces was drawn at one bleeding from the arm, and 20 ounces more, very soon after, from the temples by cupping.

The object of Chapters XII. and XIII. is, in the first, to illustrate the rapidity with which a fatal issue takes place in some cases of Scarlatina Maligna; and, in the second, to suggest how far the common practice among English medical men, of commencing the treatment of this, and of almost all other fevers, with a purgative, does not often tend to aggravate the symptoms and accelerate the death of the patient. We think him quite right in this particular. Indiscriminate active purgation in Fevers of a bad type, and more especially in the Exanthemata, has unquestionably produced a great deal of mischief. The older physicians very generally condemned the use of purgatives at the commencement of malignant febrile diseases. As a general rule, it would be much safer and better practice to substitute an emetic for the purging dose, that is now so usually prescribed under such circumstances.

Dr. Mayo seems to have a decided leaning to the use of the cold affusion in the treatment of Scarlatina, but he does not adduce any evidence in its favour. He considers it not improbable that "the deep-seated tendency of the English practitioner, always to commence the treatment of fever with aperient medicines, may have rendered the experiment of cold affusion thus far unsuccessful in fever generally;" and he thinks that "the neglect, which Dr. Curry's discovery has met with, infinitely discreditable to the medical science of England."

The chapter headed "Chronic Cerebral and Spinal Cases successfully

* Medical Writings of the late C. H. Parry, M.D., vol. i. p. 474.

treated," is surely not worthy of a place in any collection of medical cases. Take, for example, the following report, as one of the instances of the successful treatment of a chronic, spinal, or cerebral disease!

"The Rev. R. S., aged 37, of a pallid countenance, but strong, short figure, consulted me in February, 1838. He complained of inability to walk, or take any exercise, from vertigo and languor; of a fixed pain, and sense of obstruction, in the occiput, and uneasiness and numbness in the loins. His utterance was slow, and very laborious; and I ascertained from his friends that complaints which he also made of growing incapacity for business, were not exaggerated, whatever the cause might be. He was a sensible man, of an even temper. I ascertained that in early youth some evil habits of an enfeebling nature had been indulged in. He was married, without children, and lived in the country.

Sumat Pil. Hydrarg. Sub. C. gr. iij.; Pil. Galbani C. gr. v., o. n.

℞ Sp. Ammoniæ Succinat. ʒiss.; Aquæ ℥vj.; 4tam partem ter quotidie.

℞ Ext. Colocynth. C. Rhei. āā ℈j.; Ext. Hyoscyam. gr. x.; in pil. x. divis. j. vel. ij. p. r. n.

"This course was pursued, with slight modifications, to the beginning of May, and was attended by the greatest relief of all his symptoms. No change was made in the general habits of Mr. R. which could explain his cure, independently of the medical measures. I may add, that I have seen this gentleman since in the enjoyment of good health." P. 126.

The solitary case of "Meningitis, shewing only increased vascularity," related in the next Chapter is—standing as it does by itself—to say the least, very profitless. "Every day's additional experience," remarks our author, "tells us, how little we know, so far as post-mortem appearances are concerned, of the evidence of sthenic inflammation, where only increased vascularity is present, in cerebral disease; and, again, the inexpediency of a depletory system in acute mania rests on good authority."

Can we report more favourably of the contents of the next Chapter, entitled "Albuminous urine—Ascites—Paracentesis Abdominis"? In truth, we cannot. The narrative of the first case is so slovenly and imperfect that it is impossible to make anything out of it. The specific gravity of the urine in the first case is stated to have been 10: the author means, we suppose, 1,010. Then, from the mere circumstance that it was slightly albuminous, he takes it for granted the kidneys were affected with granular degeneration; and, finding that his patient appeared to be rather benefited than otherwise by the use of mild mercurials, he hints that these have been needlessly reprobated in Renal Diseases. "The opinions, expressed on high authority against the use of mercury under presumed granular disease, deserve to be reconsidered. The inflammatory process often attendant on that state is such as mercury influences curatively in other instances. The question, no doubt, must be settled by experience; but, as far as analogy is concerned, the burthen of proof rests rather with those who impugn the expediency of mercurials in these cases, than with those who may assert it."

The case of presumed "Cæcal Abscess," opening externally upon the right nates, deserves brief notice only from the strange tone of the remarks with which its history concludes:

" Besides the general interestingness of the subject, I am inclined to think that our diagnosis, both in abdominal and thoracic disease, might be helped by farther inquiry into the distinctive cerebral phenomena to which they may give occasion.

" Whatever interest the above particulars may claim, not the least important fact which they convey is the completeness of the patient's recovery under the agency of the waters of Homburg and Schwalback. And I may here observe, that the patient, whose judgment and clearness of intellect might well be relied on, has affirmed to me with great distinctiveness the benefit which she received from those waters, independently of the general good obtained from air and scene, to which we are, perhaps, disposed to attribute more than the due share which they are entitled to in the recovery of chronic cases." P. 152.

The case of "Double Consciousness, &c.," occurring in an hysterical girl, related in Chapter XVIII., leads Dr. Mayo into the dubious paths of metaphysical speculation. He shews a kindly feeling to the Mesmerists, and considers that they have been unfairly dealt with by the medical profession. " We allow our nurses," says he, "to rock our infants to sleep. Are we to be told that it is absurd and unjustifiable to produce a form of sleep, during which pain is unfelt, and irritation allayed, by movements of the hands ?" He evidently believes in the reality of the mesmeric *rapport*, and its accompanying marvels :—

" Whatever the beneficial effects of mesmerism may turn out to be, it comes before us fraught with liabilities to abuse and mischief in its application to chronic disease, which is likely to be its especial subject, of no common kind. The possession which it gives to the manipulator of the person's mind who is subjected to his agency, is of a nature to justify these fears, and there is reason to suppose that it has been sometimes turned to the worst account by unprincipled persons." P. 157.

There is no evil that we ever heard of as likely to result from mesmeric manipulations save that—a great and most mischievous one, it must be admitted—of their being made the occasion of acting upon the sexual feelings of young females. This, in short, is the secret of almost all the cases of successful mesmeric practice having occurred in unmarried women. Hence, too, its deserved reprobation by the great mass of the medical profession, as well as by the public generally, as a means of therapeutic relief. The guarded and hesitating manner, in which our author handles the subject, betrays a sad want of confidence in his own judgment; for, if we mistake not, Dr. Mayo has been making enquiries into Mesmerism for some years past. With a decided leaning to its claims—for in one passage he does not hesitate to recognise the *truthfulness* of the assertion, that Mesmerism is "a law of nature which is as much a part of the human constitution as the processes of thought and digestion" !—he will not, however, commit himself either one way or the other, but suggests that the Royal Society, or some such public body, should appoint a commission to examine into its merits.

" One would have thought, that the extremely interesting nature of the mental phenomena disclosed by mesmerism, supposing it to be truthful, or to contain truth, would have so strongly disposed philosophical men to the inquiry which I am suggesting, as that no arguments such as I am adducing should be needed. It is to be regretted that there is no royal society for psycological as well as for material phenomena.

"A candid inquiry into what may be termed the more substantial forms of empiricism is expected generally from us by the public; for the most important physical truths must have had an empirical period in their promulgation. At the same time, considerable difficulties are thrown in our way by that same public, in the execution of this duty. The public is, indeed, a severe task-master. It is at once inquisitive and sensitive in regard to so-called quackery; prompt in imputing that contumelious term to regular practitioners, if they afford attention to the class of subjects, and equally prompt in imputing to them an uncandid spirit if they decline to do so.

"Our efforts in this direction will, in truth, be most effective, and at the same time least detrimental to ourselves, if directed from without; the governments of other countries have empowered commissions of inquiry into such matters. There never has been an epoch in the history of medicine at which such a commission has been more expressly indicated than at the present moment." P. 159.

What good, may we ask, has the double French Commission done? Next to nothing; both parties, mesmerists and their opponents, have appealed to its reports in justification of their opinions.

The transition from one description of empiricism to another is just what might be expected. We were therefore not surprised to find, after Dr. Mayo's bland demeanour towards Mesmerism, that he was nearly equally courteous to the sister science (!) of Homœopathy. He alludes, at first, with expressions of admiration, to the "philosophical investigation" of its claims, which one of our contemporary Journalists has recently instituted, and the result of which has been to convince him (not Dr. M.) that the homœopathists have the merit of curing nearly as many patients as the regular practitioners! Our author has not a single word to say in the way of contradiction or of protest against this specimen of ignorant credulity. But when he is coolly told by one of this gentleman's correspondents— whose communication is declared to be "of high stamp and admirable tendency,"—that, "in all cases and on all occasions, Nature is truly the agent in the cure of the disease; and that, as she acts in accordance with fixed and invariable laws (what, pray, is the meaning of this in reference to diseases?), the aim of the physician ought always to be to facilitate her efforts, by acting in harmony with, and not in opposition to, these laws," he at once detects the fallacy of the assertion, and exposes its absurdity with considerable spirit in the following passage:—

"That diseases follow laws and a course imposed by nature is most true; this I presume to be the meaning of Dr. Combe; it is moreover equally true, that in many cases this law, or course, though it is quite compatible with the completion of the disease, is incompatible with the life of the patient. Thus, when pneumonia proceeds from its congestive to its inflammatory stage, then onward into hepatization and purulent infiltration, can anything be more natural or more fatal than its progression? As a disease in which the so-called natural treatment is applicable, that is, in which most of those resources must be excluded which thought has elicited from the stores of nature, Dr. Combe has most unhappily selected pleurisy; and here, supposing the exciting cause not to have been violent or long-continued, he recommends the absence of medicinal agents. He even argues in favour of effusion being allowed to take place. The possible slowness of its unassisted absorption, its injurious influence upon the investing membrane of the thoracic cavity, the gradual substitution of a purulent for a serous effusion, where blood-letting and the use of mercury might have forbidden that event, is left unheeded. Surely these are contingencies not to be hazarded with a view to follow laws of disease which lead to structural disturbance. The laws and history of ague have been most attentively and wisely studied for many ages; not in order that our practice might be in unison with those laws, but that it might interrupt and break the sequence of phenomena which would take place under them. If the disease be permitted to exhaust itself under this sequence, the patient is probably exhausted *pari passu.*" P. 183.

A little farther on, Dr. Mayo makes the following very just observations:—

"There is something very attractive at first sight in the prostration of scientific interference before natural laws. But it is not justified by the history of the laws of *disease,* in the extent to which it is recommended. That history and those laws must in fact be attentively perused and excogitated, as the plans of an enemy are studied, and with the same intention; namely, with a view to obviate them or prevent their development. The doctrines of non-interference may gain a temporary sway through the talents of their supporters, but this truth, habitually familiar to the English mind, will afterwards recur in full force, and 'Young Physic' will rise from a temporary suspension of active measures to a more heroic use than ever of calomel and the lancet." P. 188.

No sooner, however, has our author given expression to these remarks, than straightway he speaks of a report of cases treated on Homœopathic Principles as "a very valuable contribution," an "excellent detail!" As a specimen, he relates from the said report a case of Pneumonia, where the patient's life was evidently sacrificed to the plan of treatment by infinitesmal doses of Aconite and Phosphorus! Yet such is the system, by the adoption of which we have been told of late, by some who have put themselves forward as would-be regenerators of the science of Medicine, "homœopathists cure nearly as many patients as the regular practitioners."

On the strength of the very mischievously mismanaged case just alluded to, Dr. Mayo screws himself up to declare homœopathy a "heresy;" but the tenderness of his spirit seems at once to rebuke him for the harshness of such an expression, "which may seem inappropriate to the labours of the very respectable practitioners, by whom it has occasionally been carried out!" and he closes his remarks by predicting "that the theory of Hahnemann, and his school, is destined to melt away and disappear, or to be consolidated in a fragmentary state with the general mass of medical knowledge, so far as any portions of it can survive the test of time and experience."

Now we must frankly tell him, that it is not by such weak and washy means as he employs that the progress of rank empiricism is to be resisted, and the scientific character of our profession is to be defended against either the open assaults of mendacious quacks, or the covert underminings of false or ignorant friends within the camp. We want bolder and stouter men than him to expose and denounce the "heresy" that he alludes to. Dr. Mayo, indeed, is not likely to apostatise from the true faith; but it would not do to rely upon his strenuous assistance in defence of it. He is one of those who are neither hot nor cold; but his lukewarmness seems to proceed rather from distrust of his own judgment than from any vacillation of principle. He evidently perceives the folly of the attempt that has lately been made to bring the *niassaries* of a do-nothing practice into favour, and yet he lacks courage to condemn it with energy and decision.

He gently taps the delinquents upon the back, expressing his regret that they have gone so far. He will not join with them; but he is not quite prepared to set himself against them. It is this weak and uncertain conduct, this half halting between two opinions, this vain seeking to reconcile the claims of science with the pretensions of quackery, that we cannot abide. It is high time for men to speak out their minds with unmistakeable decision. For any one, who has had the opportunity of studying diseases not in the closet or from books alone, but by the bedside of the sick, to try and make us believe that recovery from an acute malady like Pneumonia is quite as likely to take place in the hands of an Homœopathic doctor as in those of a regular Physician, is a piece of effrontery that we were certainly not prepared to expect in the present day. But the foolish attempt will not do. There may be much to correct alike in the science and in the art of medicine in the present day, both in this country and abroad. But it is assuredly not by preaching down either the over-active practice of some physicians, or by preaching up the do-nothing sillinesses of empirics; it is not either by denouncing polypharmacy on the one hand, or by eulogising homœopathy on the other; it is not by suggesting the trial of every new remedy,* or by giving currency to the assertions of such men as Fleischmann or Henderson—alas! the disgrace to that uni-

---

* The last suggestion, that has been made by one of the disciples of the "Young Physic" school, is the use of the cold-water sheet in the treatment of Fever! Some of the cases, treated according to the Preisnitzian plan, were cured within forty-eight hours! Morrison's Pills, we doubt not, could do the same. At least, an Hygeist would tell you so. But a much more offensive communication, than even that of the surgeon of the Leicester Dispensary, is the account given by a medical officer of one of H. M. ships of the practice which he has been pursuing, of late years, in the treatment of the crew that were committed to his professional care. This person—he wisely withholds his name, and that of the vessel to which he is attached—informs us that, upon a recent outbreak of Fever (the remittent of the Mediterranean, we believe) on board his vessel, he selected twelve cases for experiment. Of these, four were treated by him with Blood-letting and the use of vigorous antiphlogistic remedies; two with large doses (a scruple) of Quinine, from the very commencement of the attack; two with liberal doses of Mercurials; two with a draught—containing 90 drops (!) of Laudanum, as many of tincture of Digitalis, and of antimonial and ipecacuan wines—to be given at once, and to be repeated in six hours; and two with mere sponging of the surface with vinegar, and the unrestrained use of water to drink. Of the first four, two died; of the next two, one died; and of the following two, one died: the others recovered. "Need I add," says this reckless promulgator of his own shame, "that, since that period, the patient labouring under Fever, who has been solely under my charge [his colleagues, we suppose, would never go along with him] has never been bled, or that but very little medicine, in the treatment of either the Mediterranean or the W. Indian Fever, has been expended in my practice." This wiseacre goes on to tell us that, for some years past, he has never used the lancet but once, on board his ship; and he closes his communication with cases of Gastrodynia, obstinate Constipation, and of Dysentery accompanied with Tape-worm, in which a cure was effected with bread-pills, after the ordinary remedies had failed! All this is certainly very creditable to the naval medical service, in the present day; and not less so to the character of professional journalism, in the 19th century!

versity which has had a Cullen and a Gregory among its teachers, and is still graced by the names of an Alison and a Christison;—it is not by such means as these, that the errors of the healing art in the present day are to be corrected. No. It is by the inculcation of a more sedulous observation of diseases and of their symptoms, by a more minute enquiry into their predisposing and exciting causes, by a more exact scrutiny of the condition of every part of the body, fluid as well as solid, internal as well as external, and by a more diligent examination of all the excretions, but not an exclusive attention to any one of them; it is by carefully observing what things do harm and what do good, and by remembering how much may often be effected by regulation of the food and drink which a patient takes—a wide field of therapeutic research—of the air which he breathes, the locality where he resides; it is by studying the operation of Mind and its thousand feelings upon the health of the Body, and of the influence of evil habits, unrestrained indulgencies, vicious practices on the physical constitution of our nature;—it is in this way, and in this way alone, that the practice of Medicine can be improved, and our profession made to maintain that place in the estimation of the wise and the good, which it was designed to occupy. The natural, and indeed inevitable, result of following such a plan will be, that medical men will be simpler in their treatment of most diseases, more scientific and less mechanical and routinist in the selection of their remedies.

Many maladies, we all know, will cease quite as well and almost as quickly without medicines—but not without medical treatment—as with them. The numerous cases of catarrhs, rheumatic pains, &c. which are so rife just now (February), will generally subside under the use of the most simple means, provided due attention be paid to diet, clothing, exercise, &c. He, who doctors such cases by mere physic, may unquestionably consult the interest of the chemist or the business-like appearance of his own day-book, but assuredly the patient will have no cause to thank him. Lower the diet, forbid strong meats and drinks, give diluents, enjoin warm clothing, and all will speedily be well;—more speedily perhaps, if some mild diuretic febrifuge be given at the same time. We have daily occasion to regret the too frequent neglect of hygienic, and the undue attention that is paid to medicinal, therapeutics. The physician, who does not give exact instructions as to the former, in almost every case that he is consulted upon, has done but one-half—and that often the least important—of his duty. In the treatment of some maladies, indeed, the direction of the regimen is almost every thing. Scrofula, in its hundred forms, bears witness of this. There is therefore quite as much skill required in the management of the diet, &c. as in the selection of the appropriate medicinal remedies to be employed. Nor are we to suppose that the treatment of diseases more by hygienic, and less by pharmaceutic, prescriptions affords any argument in favour of the extravagant notion that, because Nature is the agent in the cure of diseases, and as she acts in accordance with fixed and invariable laws, therefore "the aim of the physician ought always to be to facilitate her efforts by acting in harmony with, and not in opposition to, these laws." There is just about as weak and pernicious folly in ascribing all, or nearly all, to the "vis medicatrix naturæ," as there is in neglecting its operations altogether. In some cases, there is no curative effort at all made by the system. What, pray, would become of the Chlorotic girl, if the cure of her malady was left to—nay, were it even influenced by—the suggestions of her own feelings, and the cravings of her appetite? Or, is the patient, we may ask, affected with Diabetes, led by the instinctive promptings of Nature to avoid the use of those very articles of food which will inevitably exacerbate his malady, and aggravate all his sufferings? Again, in other cases, Nature may indeed effect a cure, or rather a termination, of an exciting disease; but it will be at the inevitable induction of a most serious lesion, or perhaps at the cost of life itself. Where is the homœopathist who, if he was labouring under an attack of Iritis, threatening the loss of sight, would be satisfied with anything short of the very active remedies which every enlightened physician would at once employ? And if inflammation is seen to produce such grave effects in the case of the Iris, its consequences are not less so in other parts which are hidden from our immediate inspection. That the subjective symptoms—those, we mean, independent of auscultatory investigation—of Endocarditis, for example, will often cease under the employment of the most simple remedies, cannot be denied; but is there not more than sufficient warrant to believe that, under such circumstances, the liability to incipient organic mischief of the Heart will be tenfold greater than if the patient had been treated according to the principles, which have been explained in a preceding article of our present Number?

We should like to have pursued these remarks, and to have endeavoured to point out what are the classes or families of diseases wherein the inexperienced practitioner is apt, on the one hand, to do too much, and, on the other, to ascribe undue importance to the medicinal agents which he may have employed in their treatment. But this we cannot do just now. Suffice it therefore merely to say, that it is more especially in the management of idiopathic Fevers of all sorts, that the operations of nature ought to be watched with most sedulous and patient attention, with the view of determining the line of practice that should be adopted. Infinite mischief has been done of late years by laying down dogmatic and peremptory rules for the treatment of diseases which change with every season, and almost vary in every individual. Had the indications of Nature, derived from a most diligent observation of all the symptoms of disease, been more faithfully followed out, and had the bold pretensions of pathological anatomy and physiology been more cautiously received, we should not now have been exposed to the taunts of the empiric, nor to the still more humiliating conviction within our own minds, that the boasted discoveries of modern medical science have not taught us how to treat a large proportion of diseases a whit more ably or successfully than did many of our predecessors in the old time before us.

